# Filing of Newspaper Cuttings

**Published:** 1908-05-09

**Authors:** L. Gwillim Davis


### FILING OF NEWSPAPER CUTTINGS.

By L. GWILLIM DAVIS, M.D.(Cantab.)

An article in a recent issue of The Hospital, on the above subject, prompts the present writer to detail his method, which has been elaborated over some five or six years, and which he ventures to think is superior to the use of books for preserving cuttings in. It also solves the problem—no easy one to a practitioner with limited house-room and a small surgery annexe—of the disposition of current medical literature. These bulky weeklies, if bound half-yearly, cost an amount of space and shillings quite incommensurate with their extent of usefulness, for at least half their matter is but little needed by the practitioner, and a part of the other half is ephemeral in its nature. It is much simpler to extract only those pages which are of lasting import, and put these away in some sort of classified order; and this can be combined with the filing of cuttings, if desired, in the same file—and all this at a cost of less than a sovereign for filing from six to ten years' material.

The flat files of the Stolzenberg type are the most useful, and better and cheaper than "box" files. The writer uses the Stolzenberg system—this is not a veiled advertisement, any similar flat file will probably serve as well—and he will briefly detail his armamentarium:—

1. Half a hundred quarto files, assorted colours; cost about 14s.

2. A perforator; cost 2s. 6d., or upwards. The 5s. and 6s. ones are fitted with a gauge and box to catch the punched-out discs, and are worth the extra money.

3. A "lifter" for inserting or detaching separate sheets filed; cost 6d.

4. (For cuttings.) The rest of the sovereign may be laid out in wrapping paper, by choice brown or blue, as cuttings stand out well from such sheets; and a pot of any good paste, such as Stickphast. Paste is preferable to gum for cuttings.

There are six colours in the Stolzenberg files, and these six may be utilised for different branches of study—*e.g.* Medicine, Surgery, Pathology, Obstetrics, etc. This makes reference very easy.

I classify my cuttings and pages thus: Diseases of Lungs and Bronchi, Diseases of Heart, and so on, having some twenty-five files. Then I have also these general subjects, each under separate file: Surgery, Anæsthetics, Fractures and Dislocations, Pathology, Therapeutics, Legal and Ethical, New Drugs (contains various leaflets, etc., sometimes useful, more often not), New Instruments Obituaries and Biographies, Miscellaneous. At the end of most of these files are a few sheets of wrapping paper for cuttings on the subject of the file; these can be added to by means of the lifter.

In extracting the article to be filed, one must see that its margins are extra broad, to allow for the space used up by the filing-prongs; and this is the only weak spot in the system, for sometimes—though in practice not often—one encroaches upon another desired article in cutting these margins. The broad margins allow of the file being used book-fashion, and the article being read right up to the end of the line. Sometimes, too, an article will follow or precede another article to be filed, when it is well to make—on a sheet of paper—a brief abstract of the part not filed, with a reference, and file it with the rest of the article. This necessity, too, does not often arise. In taking out cuttings, each should be dated, and a rubber dating-stamp is convenient for this. In pasting in cuttings, it is sufficient to paste the inner edge only of the cutting.

It will happen in time that in some file matter will accumulate. It will then be desirable to consider whether some of this should not be extracted with the "lifter" and put into another file. Thus my file on the "Alimentary Canal" has now expanded into: (1) Stomach and Small Intestine, (2) Large Intestine, (3) Abdomen Generally, (4) Appendicitis. Observe that the system is practically self-indexing—*e.g.*, one gets all the papers on Duodenal Ulcer for years past in file (1) instead of looking them up in several volumes of journals. Occasionally one may have to cross-reference—*e.g.* "Empyæmata in Children" would probably go into the file for Diseases of Children, with a sheet in the Lung file indicating this, or a note made on the cover of the latter file.

The foregoing sounds involved and laborious; it is really simplicity, at the cost of a minimum of work, if only it be done once a week or so and no arrears allowed to accumulate. Fifteen minutes to go through the journal to be filed, having previously extracted its metal clips with a pliers or screwdriver—another fifteen or twenty minutes next day to file away the matter—*voilà tout.* I would repeat that the margins of the filed pages must be extra broad, to allow of turning well over and reading to the end of the lines; and the more files used, the better the self-indexing.

Though not essential, a filing cabinet—cost about £3—is a great comfort; but whether used or not, let the files be put away *flat.* From six to eight shelves about four inches deep will be sufficient, and a jobbing carpenter will put them up for a small sum. The shelves should be scolloped out in the middle, to allow of easy handling of the files.

